# First Language Matters: Event-Related Potentials Show Crosslinguistic Influence on the Processing of Placement Verb Semantics

**DOI:** 10.3389/fpsyg.2022.815801

**Published:** 2022-07-07

**Authors:** Annika Andersson, Marianne Gullberg

**Affiliations:** ^1^Department of Swedish, Linnaeus University, Växjö, Sweden; ^2^Centre for Languages and Literature, Lund University, Lund, Sweden

**Keywords:** semantic processing, crosslinguistic influence (CLI), ERP, second language processing, N400, P600, placement verbs

## Abstract

Second language (L2) learners experience challenges when word meanings differ across L1 and L2, and often display crosslinguistic influence (CLI) in speech production. In contrast, studies of online comprehension show more mixed results. Therefore, this study explored how L2 learners process fine-grained L2 verb semantics in the domain of caused motion (placement) and specifically the impact of having similar vs. non-similar semantics in the L1 and L2. Specifically, we examined English (20) and German (21) L2 learners of Swedish and native Swedish speakers (16) and their online neurophysiological processing and offline appropriateness ratings of three Swedish placement verbs obligatory for placement supported from below: *sätta* “set,” *ställa “*stand,” and *lägga “*lay.” The learners’ L1s differed from Swedish in that their placement verbs either shared or did not share semantic characteristics with the target language. English has a general placement verb *put*, whereas German has specific verbs similar but not identical to Swedish, *stellen* “set/stand” and *legen* “lay.” Event-related potentials (ERPs) were recorded while participants watched still frames (images) of objects being placed on a table and listened to sentences describing the event with verbs that either matched the image or not. Participants also performed an offline appropriateness rating task. Both tasks suggested CLI. English learners’ appropriateness ratings of atypical verb use differed from those of both native Swedish speakers’ and German learners, with no difference in the latter pair. Similarly, German learners’ ERP effects were more similar to those of the native Swedish speakers (increased lateral negativity to atypical verb use) than to those of the English learners (increased positivity to atypical verb use). The results of this explorative study thus suggest CLI both offline and online with similarity between L1 and L2 indicating more similar processing and judgments, in line with previous production findings, but in contrast to previous ERP work on semantic L2 processing.

## Introduction

Languages differ in what meanings they express. For example, to describe the simple placement of objects on a surface, speakers of languages like Swedish and German have to choose from sets of mandatory placement verbs: *sätta* “set,” *ställa* “stand” and *lägga* “lay” in Swedish and *stellen* “set/stand” and “*legen* lay” in German (e.g., Kutscher and Schultze-Berndt, 2007; Gullberg and Burenhult, 2012). In contrast, English speakers use a general placement verb *put* for all sorts of placement, even though English technically also offers fine-grained verbs such as *sit*, *stand*, and *lay* (David, 2003; Gullberg, 2009). Importantly, such cross-linguistic differences in the selection of relevant semantic information and form-meaning mappings raise important challenges for second language (L2) learners as they start to use a new language with different semantic categories or category boundaries in the same domain, often leading to crosslinguistic influence (CLI) (Jarvis and Pavlenko, 2008 for an overview). This study probes the effects on L2 lexical processing of having no corresponding category in your first language (L1) vs. having similar but not identical categories. We used event-related potentials (ERPs) to examine how German and English learners of Swedish process descriptions of object placement in L2 Swedish and specifically whether they differ in ways suggesting an influence from their L1. Crucially, German has similar but not identical verb categories to Swedish, whereas the English verb category is not similar to Swedish at all. By comparing learners’ processing to that of native speakers of Swedish, the study explored how the verbs are processed to probe whether and how a native language can affect online semantic processing in an L2.

This study aimed to extend what we know about how different vs. similar semantic categories are processed. Importantly, it also extended previous research on CLI on neurophysiological processing to the processing of semantics of shared or non-shared semantic categories. Electrophysiological studies of the effects of CLI have previously focused foremost on syntax and morphosyntax (e.g., Sabourin et al., 2006; Sabourin and Stowe, 2008; Dowens et al., 2011; Carrasco-Ortíz et al., 2017; Andersson et al., 2019; von Grebmer Zu Wolfsthurn et al., 2021, but see FitzPatrick and Indefrey, 2010; Midgley et al., 2010 for studies of cognates).

## Background

### Crosslinguistic Influence in Semantic Processing in a Second Language

Studies of L2 comprehension and production have long focused on issues of form and morphosyntax over issues of meaning and semantics. In production studies, it is often tacitly assumed that if a form in production looks “target-like,” then it also has “target-like” meaning. However, there is considerable evidence that this is not the case. For example, when carefully probed, L2 speakers turn out to attribute different meanings to spatial prepositions than native speakers of English (Ijaz, 1986), and to prenominal and postnominal adjectives in French (Coppieters, 1987). The differences found in L2 semantics seem to depend on the relationship between semantic categories in L1 and L2. It is often assumed that similarity or semantic equivalence facilitates learning whereas other types of relationships (more or less fine-grained distinctions in the L1 or the L2) raise challenges if the goal is “target-like” meaning (cf., Pavlenko, 1999). However, all transitions from an L1 to an L2 potentially require restructuring of meaning in the L2, involving a range of possible processes such as shifting of semantic boundaries, the creation of new categories, or the splitting of existing categories (Stockwell et al., 1965; and cf. Ellis, 1994; Pavlenko, 1999).

It seems to be particularly demanding to move from a single-term system in the L1 to a more fine-grained, multiple-term system in the L2 (cf. Stockwell et al., 1965; Ellis, 1994). For example, in production, English learners of L2 Spanish are challenged by the need to distinguish permanent and temporary states of being (*ser* and *estar, “*to be”; Geeslin, 2003), presumably since they must introduce a semantic distinction in a domain where their L1 does not make one. Similarly, it can be taxing for English learners of L2 Russian to distinguish *zlit’sia* (to feel anger in general) from *serdit’sia* (to be actively cross, angry, mad at someone in particular) (Pavlenko and Driagina, 2007). Again, introducing semantic distinctions in an L2 domain where they are lacking in the L1 is challenging. In production, these challenges are often dealt with through overgeneralization and simplification.

Moving in the opposite direction, from a language with multiple distinctions in a given domain in the L1 to an L2 with a single category, has received much less attention both in production and in comprehension studies. Semantic differences in the L2 are harder to detect when the surface form is underspecified relative to meanings in the L1. However, there is some evidence suggesting that this transition also requires semantic restructuring if the meaning is to be “target-like.” For example, Dutch L2 learners of English readily use the term *put* but their manual gestures, which encode meaning, suggest that they often continue to operate with more fine-grained meaning from their L1 where two terms, *zetten* “set” and *leggen* “lay” distinguish object properties (Gullberg, 2009).

Whereas production studies thus indicate that semantic reorganization is challenging and that L2 semantics may not be “target-like” even when L2 forms appear to be, it is less well known whether comprehension, and especially online comprehension processing, is affected in the same way. The vast literature on cognate processing in L2 users and bilinguals (using terms such as interlingual homographs, interlingual homophones, or cognates) largely shows facilitatory effects on processing for cognates (see Lijewska, 2020 for a recent overview). Although these effects are not typically construed as CLI but are rather discussed in terms of language-selectivity in lexical access (or a lack thereof), they indicate that similarity facilitates processing. In contrast, the so-called false cognates or false friends (similar form, but different meaning) render processing more effortful (although that may depend on the task; Marecka et al., 2021). These effects of course resonate with the literature on CLI where facilitatory effects have traditionally been referred to as positive transfer/CLI and other effects as interference (cf. Jarvis and Pavlenko, 2008 for an overview). However, these studies do not typically delve into the details of differences in semantics beyond a simple difference in congruency across languages, nor do they consider effects beyond the single word, such as effects of semantics situated in utterances. An exception to this is a study using eye-tracking and a visual world paradigm to examine the online processing of Dutch placement verbs. The study investigated whether L2 learners of Dutch showed anticipatory eye movements to objects while listening to Dutch placement event descriptions (van Bergen and Flecken, 2017). The L1s of the learner groups differed in the degree of similarity to Dutch: German (similar to Dutch), English, and French (not similar to Dutch). The results showed that German L2 listeners, like native Dutch listeners, anticipated objects that matched the verbally encoded position immediately on encountering the verb. French and English L2 participants, however, did not, suggesting that shared semantic contrasts facilitate prediction.

Overall, despite the large literature on CLI in production and on cognate processing in comprehension, we still know relatively little about the online effects of crosslinguistic differences in L2 fine-grained semantic processing as a function of the L1. The examination of this issue requires a semantic domain where there are robust crosslinguistic differences. In the current study, we will probe the domain of caused motion, specifically placement, which we introduce in more detail below. Moreover, by combining acceptability ratings with temporally sensitive neurophysiological measures, it is possible to investigate whether processing differs in kind and also in timing in relation to an L1. Below, we will give a background on neurophysiological studies using ERPs to investigate semantic processing in L1 and L2, a well-studied field even if there is an apparent lack of studies of CLI using this methodology. We will also present ERP studies of event processing since placement could be construed as an event, which is therefore relevant for the current study.

### The Semantic Domain of Placement

In the domain of placement, there are considerable crosslinguistic differences (Bowerman et al., 2004; Narasimhan et al., 2012). Some languages use a single general term. English, for instance, uses *put* for all placement on a surface with support from below. This simple verb lacks a direct translation in many of the world’s languages (Ameka and Levinson, 2007), which instead use bigger or smaller sets of obligatory verbs, making more fine-grained semantic distinctions, and which may or may not have a superordinate term that is equivalent to *put*. Swedish, for example, has three mandatory placement verbs to describe the placement of objects with support from below: *sätta, ställa*, and *lägga “*set,” “stand,” and “lay.” The choice of verb depends on the object’s orientation (horizontal/vertical) and properties such as being symmetric/asymmetric (e.g., cube/candle) and being with or without a base (e.g., cube/ball) (Gullberg and Burenhult, 2012). The most frequent verb *sätta* “set” is typically used to describe an object made to rest on its base in a canonical upright position (e.g., a bowl placed on a table). The verb *ställa* is similarly used but especially with vertically extended objects (e.g., a bottle). *Lägga*, in contrast, is typically used when an object is placed off its base or when it lacks a base (e.g., a bottle placed lying down or a ball) (Gullberg and Burenhult, 2012). Languages like Dutch and German operate with similar small sets of placement verbs. Other languages have much wider repertoires with specific classificatory placements of verbs for different objects (long, thin, round, etc.), such as in Mayan languages (cf. Narasimhan et al., 2012).

Although the placement verbs are highly frequent in production, they are challenging to acquire for children (Toivonen, 1997; Hansson and Bruce, 2002; Narasimhan and Gullberg, 2011) and L2 learners (Viberg, 1998, 1999; Lemmens and Perrez, 2010; De Knop and Perrez, 2014). For instance, Dutch children acquiring two obligatory placement verbs differ from adults in descriptions of the same placement scenes as late as age five, often using only one of the two obligatory verbs to refer to all placement scenes (Narasimhan and Gullberg, 2011). In L2 acquisition, the L1 has been shown to influence the L2 production of placement verbs. For example, Polish L2 learners of Swedish, whose L1 has overlapping but not identical categories to Swedish, use verbs more similarly to Swedes than speakers of Spanish and Finnish whose L1s have only a single superordinate placement term (Viberg, 1999). In a study of English L2 learners of Dutch, which also has a set of obligatory placement verbs, *zetten* “set,” and *leggen* “lay,” learners dealt with the challenge of making more semantic distinctions by using simplification and avoidance strategies, often producing dummy verbs such as *doen* “do” instead of target forms (Gullberg, 2009). Interestingly, when “target-like” verb forms were used, they still often conveyed L1-like meaning, as reflected in L1-like gestural patterns.

Given these crosslinguistic differences and findings for production, placement makes for an interesting test domain for CLI in L2 online semantic processing.

### Neurophysiological Processing of Semantics

In one of the earliest studies of language processing, Kutas and Hillyard (1980) observed a semantically sensitive ERP component, the N400. This component is observed as an increased negative deflection for words not expected in the semantic context (i.e., incongruent words) compared to words that are (i.e., congruent words). For instance, the N400 becomes stronger in amplitude in response to the word *cry* when it is presented in a context where it is not expected (e.g., *The pizza was too hot to cry*; Kutas and Hillyard, 1980). The N400 is most often distributed medially and centro-parietally for auditory and visual presentation in both L1 and L2 (e.g., Kutas and Hillyard, 1980; Ardal et al., 1990; Mills et al., 1993; Weber-Fox and Neville, 1996, 2001; Neville et al., 1997; Osterhout and Nicol, 1999; Brown et al., 2000; Hahne, 2001; Ojima et al., 2005; Osterhout et al., 2008; Newman et al., 2012). It can last longer and be of smaller amplitude in L2 (Ardal et al., 1990; Hahne, 2001; Hahne and Friederici, 2001; Weber-Fox and Neville, 2001), an effect that has been found to be related to formal proficiency rather than the age of acquisition (Holcomb et al., 1992; Hahne, 2001; Hahne and Friederici, 2001; Weber-Fox et al., 2003; Moreno and Kutas, 2005; Ojima et al., 2010; Elgort et al., 2015).

Although the N400 has been widely studied in relation to semantic processing and retrieval of lexical-semantic information, more recent studies have also described a centro-parietal positivity, the P600, for semantic processing. For example, following the sentence *Bill jumped in the lake. He made a big*…, the P600 has been reported for words that are anomalous and related (*mermaid*) or unrelated (*guide*) to the expected word (*splash*) (DeLong and Kutas, 2020). This positivity has been elicited in L1 and L2 and is generally thought to reflect difficulties with integration, including the difficulty introduced by phrase structure violations (Osterhout and Holcomb, 1992, 1993; Osterhout et al., 1994; Kaan et al., 2000; Kaan and Swaab, 2003; White et al., 2012). In reference to semantic processing, the P600 has been reported mainly when there has been a close semantic relationship between the verb (e.g., *devouring*; Kim and Osterhout, 2005) and its preceding arguments (*The hearty meal was*…) in terms of plausibility (Kolk et al., 2003; Kim and Osterhout, 2005; Kuperberg et al., 2007). In the example, the verb is related to the noun but seems to suggest a thematic role of the Agent rather than the expected Patient. Moreover, in cases where a task is included such as appropriateness ratings, the P600 has been found to reflect a reanalysis of the sentence or the word in studies of semantics (Van Petten and Luka, 2012; Brouwer and Crocker, 2017).

A few ERP studies focus on the processing of semantics in events or actions. Although these have not investigated processing in relation to language *per se*, they are still relevant for the processing of placement events. These studies have presented participants with consecutive still frames (images) to probe violations of expectations by manipulating the instrument or function (Balconi and Caldiroli, 2011), the orientation of the instrument or function (Bach et al., 2009), and the action goal (West and Holcomb, 2002). Bach et al. (2009) presented participants with two sequential images, one of an instrument (e.g., a key) followed by another image of an object (e.g., a keyhole). In one context, the instrument was functionally the correct instrument to use for the object but oriented in a way that would prevent a successful action (e.g., a hand holding a key at a horizontal angle not matching a vertical keyhole presented on the subsequent image). In the other context, the instrument violated the function, presenting an instrument in relation to an unexpected event (e.g., a screwdriver followed by an image of a keyhole). Participants viewed instruments that were followed either by objects that either matched or mismatched orientation, function, or both. All violation types (mismatches in orientation and/or function) elicited the two ERP effects, the N400 and the P600. Interestingly, the P600 was similar across types of mismatch while the N400 differed in distribution over the scalp with the type of mismatch, which could be due to an overlap of the two effects. Accordingly, differences in the N400 amplitude could reflect differences in certainty, which affects the P600 amplitude, rather than prediction, which affects the N400 amplitude (Dröge et al., 2016). As such, the centro-medial and longer-lasting N400 for object violations in comparison to orientation violations could indicate that participants did not have to reanalyze and were sure of their ratings when an odd object was used, whereas the choice was more difficult when the object was correct but the orientation was odd.

In the more complex presentation of an event, four sequential images were presented. Balconi and Caldiroli (2011) presented participants with three consecutive images building up an expectation of action. The final fourth image could either be congruent with the expectation or be incongruent with it by violating the expected goal or reaching the goal, but in an atypical way. An example of a violation of the action goal is three images of a woman and a screwdriver that is picked up, followed by the final and critical image where the woman is brushing her teeth with the screwdriver. An example of the instrument being used in an atypical way is three images of the woman with a bottle, a plate, and a glass where the final and critical image is the woman pouring water from the bottle onto the plate. As in the previous study, these violations were connected to an increased N400 as an indication of incongruency with expectation. Further, as in Bach et al. (2009), the distribution of the N400 was affected by the type of violation such that an atypical action was related to a more posterior (temporoparietal) effect in comparison to the effect for violation of the expected goal (frontal). This frontal distribution replicated previous studies of processing images violating the expectation (e.g., West and Holcomb, 2002).

In addition to highlighting the importance of controlling for effects of visual presentations to objects in unexpected orientations, these studies showed that the processing of semantics and of events can affect the amplitude and onset of both the N400 and P600. However, there is an apparent lack of knowledge of the processing of events that are guided by language, such as placement events where languages differ in the use of placement verbs. In addition, we know little about whether differences in how these events are described in an L1 affect how they are processed in an L2, that is, whether there are CLI effects in semantic L2 processing. On the whole, ERP studies of CLI have mainly been occupied with morphosyntax (Tokowicz and MacWhinney, 2005; Sabourin et al., 2006; Sabourin and Stowe, 2008; Dowens et al., 2010, 2011; Banon et al., 2018; von Grebmer Zu Wolfsthurn et al., 2021, but see Novitskiy et al., 2019 for processing of cognates, and Tolentino and Tokowicz, 2011 for a review). Also, previous ERP studies show no differences in how the brain processes L2 compared to L1 semantics (Ardal et al., 1990; Mills et al., 1993; Brown et al., 2000). However, these studies have not examined crosslinguistic differences in semantics but rather cases where the semantics are assumed to be equivalent across languages, such as in nouns appearing in a congruent or incongruent sentence context (e.g., *he spread the warm bread with socks*, Kutas and Hillyard, 1980). These studies have also typically only examined learners from one L1, meaning that it is not clear whether differences in L2 processing are attributable to CLI or to a general learner behavior (cf. Jarvis, 2000).

### Placement Verb Semantics—The Current Study

The current study explored the potential effects of CLI in the L2 acquisition of placement verbs in Swedish. We investigated two groups of L2 learners of Swedish, whose L1 placement verbs either do (German) or do not (English) share semantic characteristics with Swedish. The German learners’ L1 placement verbs were somewhat similar to the Swedish placement verbs (*sätta, ställa, lägga*) in that they form a set in which an important semantic characteristic is related to the properties of the object being placed (Kutscher and Schultze-Berndt, 2007; Gullberg and Burenhult, 2012). In contrast, the English learners’ L1 provided a single English general placement verb *put*, which does not share semantic characteristics with Swedish since it does not care about object properties the way the Swedish placement verbs do. Although the specific Swedish verbs have cognates in all three languages (*ställa-stellen-stand, sätta-setzen-set, lägga-legen-lay*), they are of very low frequency in English compared to Swedish and German (David, 2003; Gullberg, 2009) where they are obligatory and high-frequency verbs.

This study, therefore, asks the following question: Do German and English learners of Swedish process placement verb semantics similarly to native speakers of Swedish or do they show effects of their L1 placement verb semantics? Specifically, do German learners, whose L1 has similar categories to Swedish, process Swedish placement verbs more similarly to Swedes than English learners, whose L1 does not? To address this question, we asked participants to watch still images of placement events while they listened to auditory descriptions of those events where the verb was either typical of native speaker usage or not, as described above. While participants watched and listened, ERPs were recorded. Following this task, participants completed an offline computerized appropriateness judgment task in which they performed metalinguistic judgments as to whether the verb used to describe a scene was appropriate or not.

Based on previous research, we expected differences in offline appropriateness ratings even in learner groups matched on formal L2 proficiency, since formal proficiency measures rarely test for fine-grained semantic understanding (e.g., Dijkstra and van Heuven, 2002; Costa et al., 2005; Hoshino and Kroll, 2008; Poarch and van Hell, 2012). Appropriateness ratings were used as predictors of ERP difference amplitudes. Similarly to previous studies recording behavioral responses in combination with the sensitive neurophysiological responses (ERPs), we expected to see different ERP responses in the groups *even* if there were no visible differences in the behavioral measures (Weber-Fox and Neville, 1996; McLaughlin et al., 2004; Coch et al., 2005).

Since the processing of fine-grained verb semantics has not been investigated with ERPs before, our predictions concerning the online processing are tentative and must necessarily draw on previous CLI studies of morphosyntax. Therefore, in this exploratory study, we attempted to find differences between groups in the online processing of placement verb semantics as a function of semantic similarity between the L1 and the L2. Importantly, the inclusion of two learner groups with different L1 distinctions that are otherwise matched on formal proficiency and age of acquisition is vital for that triangulation to be made.

## Materials and Methods

### Participants

We recruited 63 participants at Lund University (excluding students of linguistics), across three groups: 16 native speakers of Swedish, 21 German learners of Swedish, and 20 English learners of Swedish.

Based on a previous study of word order processing comparing the same three groups, we expected an effect size of 0.05 (η*_*p*_*^2^, *f* = 0.23 the effect size measure used in G*power). We used G*power (Faul et al., 2009) to calculate the sample size for three groups (Swedish native speakers, German learners of Swedish, English learners of Swedish) by three verbs (*sätta/ställa/lägga*) for two measurements (typical/atypical). It indicated that a sample size of 117 was needed (alpha level l0.05). However, the final sample only consisted of 57 participants distributed across three groups (Table 1). The limited number of participants was due to restrictions during the COVID-19 pandemic. As a consequence, the results from this study are more exploratory in nature than originally planned.

**TABLE 1:** Group demographics.

	*N* (F)	Age (*SD*) range	SES (*SD*) range	AoA (*SD*) range	Exposure (*SD*) range	Swedex (*SD*) range
Swedish	16 (7)	30;4 (7;8) 19–41	5.6 (1.2) 3–7	n.a.	n.a.	n.a.
German	21 (14)	33;2 (6;4) 21–43	4.3 (2.1) 2–7	23;11 (3;7) 18–32	9;0 (5;1) 1–21	9.35 (0.72) 7.75–10.00
English	20 (14)	33;6 (5;10) 24–42	5.4 (1.3) 3–7	24;11 (4;3) 19–33	8;5 (5;10) 1–23	9.34 (0.79) 7.50–10.00

*Age in years; months. means given with standard deviations within brackets except for the first column where number of females are given. Socioeconomic status (SES) given as the proxy maternal education on a seven-point scale (Hollingshead, 1975) included (1) less than 7 years of education, (2) between 7 and 9 years of education, (3) 10–11 years of education (part of high school), (4) high school graduate, (5) 1–3 years at college (also business school), (6) 4-year college graduate (BA, BS, BM), and (7) a professional degree (e.g., MA, MS, ME, MD, PhD). Age of acquisition (AoA) given in years; months. Exposure calculated as age minus AoA given in years; months. Language proficiency measured with Swedex B1 (vocabulary and grammar, 40 tokens which earned 0.25 points each, thus max 10.00 points). n.a. refers to not applicable as AoA is zero, exposure is identical with age, and this group did not take the language proficiency test.*

Participants filled in three questionnaires targeting language background (Gullberg and Indefrey, 2003), handedness (Oldfield, 1971), and socioeconomic status (SES; Hollingshead, 1975). The latter was included because SES background has been shown to be related to language proficiency (Bornstein et al., 2003; Hoff, 2003a,b, 2006) and to differences in language processing (Pakulak and Neville, 2010), and we wanted to ensure that the learner groups were matched on this variable. Learners of Swedish completed the Word and Grammar subtest of Swedex, a standardized Swedish proficiency test for L2 learners (Swedex, 2012). This test targets level B1 of the Common European Framework of Reference for Languages (Council of Europe, 2001), i.e., an intermediate level of proficiency.^1^ Two English learners were excluded due to low scores on Swedex (below 5, i.e., less than 50% correct on the test). Four Swedish native speakers were excluded for reasons of age (for age effects on ERP, see Wlotko et al., 2010), early simultaneous bilingualism, and technical malfunctions. The remaining participants (*N* = 57) had normal or corrected to normal vision, reported normal hearing, and had no history of neurological or language disorders. Table 1 summarizes the participant characteristics.

All groups were matched on age [*F*(2, 54) = 1.22, *p* = 0.303]. An interaction with SES and group [*F*(2, 54) = 3.16, *p* = 0.05] was driven by German learners having slightly lower but within the same mid-SES ranges as Swedish native speakers [*t*(35) = 2.10, *p* < 0.05] (thus, we did not expect any differences in language processing in relation to their SES). However, importantly, the two learner groups were matched on SES [*t*(39) = -1. 85, *p* = 0.073, corrected for unequal variances], AoA (*t* < 1), length of exposure (*t* < 1), and proficiency (*t* < 1).

### Materials and Testing

#### Event Stimuli

The event stimuli consisted of images of placement events and auditory descriptions of the same events. We manipulated whether the placement verb used was typical of native speaker usage or not (typicality: typical/atypical) depending on the object shape (with/without base; symmetric/asymmetric) and orientation against the ground (horizontal/vertical for objects with a base).

**FIGURE 1:**
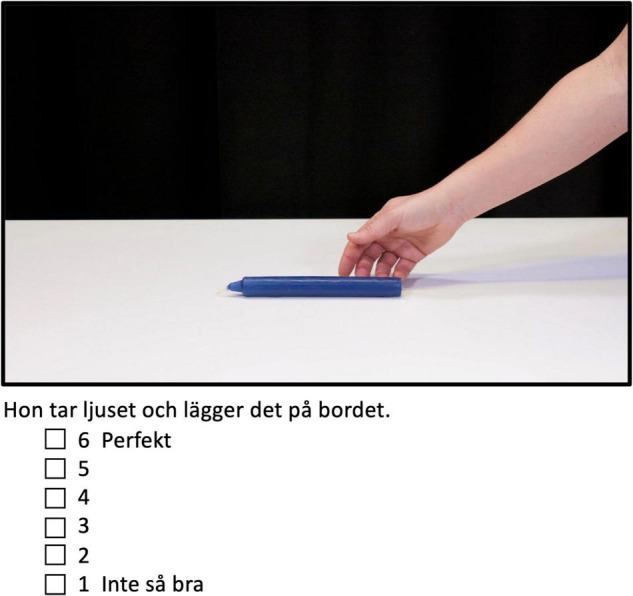
Appropriateness ratings of how well the verb fits the depicted event. Participants rated the sentence, such as in the example above; *Hon tar ljuset och lägger det på bordet* “She takes candle.DET and lays it on table.DET” by ticking the box next to the numbers from 1 *Inte så bra* “Not so good” to 6 *Perfekt “*Perfect” in an untimed questionnaire.

More specifically, the images depicted a placement event, such as a glass in a horizontal position on a table with a hand in the background (cf. Figure 1), where the hand shape was constant across all objects to avoid that it reflected the properties of the object being placed (Gullberg, 2009, 2011). Objects in the experimental still frames differed in shape and orientation against the ground. Eighty experimental pictures were constructed (see Table 2 and Supplementary Appendix A for a full list). A further 111 fillers were added consisting of vertically or horizontally oriented asymmetric objects in positions, one of which could be considered to be unusual (refer to Table 2 and Supplementary Appendix A). This included an avocado horizontally placed and a key vertically placed balancing on its edge. Shapes of animate and inanimate fillers included having legs (e.g., a symmetric pillow in the shape of an angry bird, a table, and a Barbie). Identical still frames were used for offline appropriateness ratings and the ERP session.

**TABLE 2:** Items presented in images.

		Symmetric		Asymmetric		Presentations
				Vertical	Horizontal	
Experimental	With base	20		20	20	60
	Without base	20		20	20	60
Total		40		40	40	120
Fillers	Odd position			16	16	32
		On legs	Off legs	On legs	Off legs	
	Animate	6	6	15 or 14	14 or 15	41
	Inanimate	8	8	11	11	38
Total		14	14	40 or 41	40 or 41	111

*Presentations refers to number of presentations of each item.*

The 231 sentences describing the placement scenes (experimental items as well as fillers, see Supplementary Appendix A) were presented in the present tense (*sätter, ställer*, and *lägger*) to keep syllable structure and word length comparable. The target clause was preceded by a contextual clause: *Hon tar × och × den/det på bordet “*She takes X.DET [the object on the picture] and X:s [one of three placement verbs] it [*den*/*det* depending on the gender of the object] on the table.DET.”

A pseudo-randomized list was generated such that the three verbs were used approximately equally often (two of the verbs were presented 77 times and one 78 times in each list, but each verb appeared equally many times across the three lists). In a list, each verb could not appear more than three times directly after each other, and no more than three typical or atypical combinations with placement verbs and placement events could appear in sequence. From this list, two more lists were generated such that each still frame was presented with each placement verb across participants.

**FIGURE 2:**
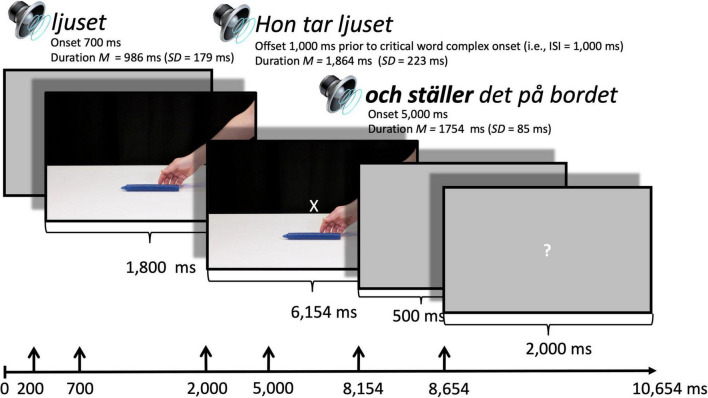
ERP paradigm. A gray screen was presented for 200 ms after which the still frame filled the screen. A sound file with the name of the object was presented such that it ended at 2,000 ms after the gray screen appeared. A cross hair appeared on top of the still frame with the first clause sound file illustrated here with *Hon tar ljuset* “She takes candle.DET.” This sound file ended 1,000 ms (ISI) prior to the second clause which started with the critical word complex here visualized in bold (onsets at about 700 ms depending on the differing length of the sound files). The second clause of the sentence started after 5,000 ms. Following the end of the second sound file (ranging 1,615–1,840 ms), a gray screen appeared, and after a subsequent 500 ms, a question mark appeared to indicate that the participant should press the red or green button to indicate if the sentence described the event in an appropriate or not so appropriate manner.

The sentences from all three lists were recorded in an anechoic chamber at Lund University Humanities Lab with a trained speaker with an accent from the region of Stockholm. From these sentences, one lead-in clause per object was chosen and spliced out using Praat software (Boersma and Weenink, 2004); e.g., *Hon tar ljuset* “She takes candle.DET”) and two clauses per verb, one for each gender, common or neuter (e.g., *och lägger den på bordet* and *och lägger det på bordet*, “and lays it [common/neuter gender] on table.DET”). Thus, the lead-in clause was held constant for each object, and the second clause was held constant for each gender and verb. Since the conjunction *och* “and” is reduced to a vowel /o/in regular speech and there is no pause between this and the verb (pronounced *o-lägger*), we chose to splice the phrases before the vowel and time lock the ERPs to this vowel rather than to the verb. The two sound files were presented with an ISI of 1,000 ms, a pause length that sounded natural and reduced risks of effects to differences in voice quality across phrases (Figure 2).

#### Appropriateness Rating Stimuli

The experimental items from the event stimuli were also used in a computerized offline appropriateness rating task (Google Docs) in which participants had to judge how appropriate the verb was for describing the placement event depicted on a 6-point Likert-scale with 1 indicating the least appropriate and 6 the most appropriate (for an example see Figure 1 and for more information regarding lists see section “Event-Related Potential Paradigm”).

Results of this untimed task were used in correlational analyses when exploring the amplitude of the ERP effects and for comparisons of the two learner groups and with native speakers of Swedish.

#### Event-Related Potential Paradigm

The participants first viewed the event stimuli. Since some objects and object names were expected to be unfamiliar (e.g., kiwano), each trial started with the presentation of a still frame with the object name (cf. Figure 2). The object name was spliced out of the lead-in clause (lengths ranging from 737 ms *bollen “*ball.DET” to 1,408 ms *hushållsrullen “*roll-of-paper-towels.DET”). This procedure was intended to reduce any N400 effects elicited by an unfamiliar object or object name during the ERP recordings of the experimental sentences. The still frame was presented for 1,800 ms after which a cross-hair appeared on top of the object in the still frame. Thus, any effects on an object in an unexpected orientation would not be included in the ERPs to the critical word (cf. Bach et al., 2009; Balconi and Caldiroli, 2011). Participants were asked to focus on the cross-hair to reduce any extensive eye movements while still permitting participants to see the placement event at a target while listening to the description. After each trial, participants made a forced binary choice “green” or “red” (counterbalancing side of the green button, right or left, across participants), indicating if they found the verb to describe the scene appropriately or not so well. This task was given to ensure that participants stayed on task, but these data were not analyzed. Ratings for analysis were instead collected in the appropriateness rating test (see section “Appropriateness Rating Stimuli”) occurring directly after the ERP session.

#### Event-Related Potential Recordings

The EEG was recorded from 30 electrodes mounted in an elastic cap (EASYCAP). Data from 10 pairs of lateral sites (F7/8, FT7/8, T7/8, TP7/8, P7/8, F3/4, FC3/4, C3/4, CP3/4, and P3/4) were included in analyses while FP1/2, O1/2 and the six midline sites (FZ, FCZ, CZ, CPZ, PZ, and OZ) were only used for detecting artifacts. This was also the case for the four additional electrodes that monitored blinks (above and below the left eye, i.e., VEOG) and eye movements (at the outer canthi of both eyes, i.e., HEOG). These electrodes had an impedance maintained below 10 kΩ, while the impedance of all other electrodes was maintained below 5 kΩ. Neuroscan SynAmps2 (bandpass 0.05–100 Hz) was used to amplify the EEG that was digitized at a sampling rate of 500 Hz. Each scalp electrode was referenced to CZ during recording and re-referenced to the averaged mastoids during offline processing. At this time, for each participant, the ERPs that were time-locked to the second phrase with the placement verb were segmented over 1,100 ms epochs with a 100 ms prestimulus baseline at each electrode site. The ERP processing was performed with the use of EEGLAB (Delorme and Makeig, 2004).

#### Artifact Rejection

From the EEG, large artifacts containing EMG were removed after which a digital, low pass filter (40 Hz) was applied. This reduced high-frequency noise prior to the ICA analyses of the EEG (“runica” routine of EEGLAB; Delorme and Makeig, 2004). From the resulting scalp topographies and component-time series, ocular artifacts were identified and subsequently removed. This step was followed by a visual inspection for residual ocular artifacts that were planned to be manually rejected; however, none were detected.

#### Procedure

The study complied with the ethical guidelines of the Swedish Research Council. Participants were awarded two movie tickets for participation. Participants provided informed consent prior to data acquisition. After this, the EEG cap was placed on the participants’ heads and electrode impedances were manipulated while participants filled in the three questionnaires (approximately 15 min). The ERP session then started, lasting approximately 1.5 h. Directly following the ERP recording, all participants performed a computerized appropriateness judgment task. They read instructions on a computer screen prompting them to be as accurate and as fast as possible in their ratings of the sentences. They then rated two test items and could then ask questions. Following this, the experimenter started a timer as an indicator of the importance of speedy responses, and the appropriateness rating started. The two learner groups finally also completed a computerized version of the Swedish proficiency test (Swedex, 2012, approximately 10 min) on the same computer but without the timer on. In total, the complete session lasted about 2.5 h including the final debriefing.

### Analyses

#### Statistical Analyses of Behavioral Testing

Average ratings for typical and atypical verb use were calculated for each participant. A repeated-measures ANOVA with two levels of typicality (typical/atypical) as the within-subject factor and language group as the between-subject factor (Swedish/German/English) was performed. This was followed by simple contrasts (paired-samples *t*) comparing each group with each other for both typical and atypical verb use. *Typical* included responses to sentences with (1) *sätta* and *ställa* with symmetric objects with base and with asymmetric objects resting on their functional base in a vertical position and (2) with *lägga* with symmetric objects without base and with asymmetric objects resting off their functional base in a horizontal position. *Atypical* was the opposite, that is, (1) *sätta* and *ställa* with symmetric objects without base and with asymmetric objects resting off their functional-based in a horizontal position and (2) with *lägga* with symmetric objects with base and with asymmetric objects resting on their functional base in a vertical position.

#### Statistical Analyses of Event-Related Potential Effects

**FIGURE 3:**
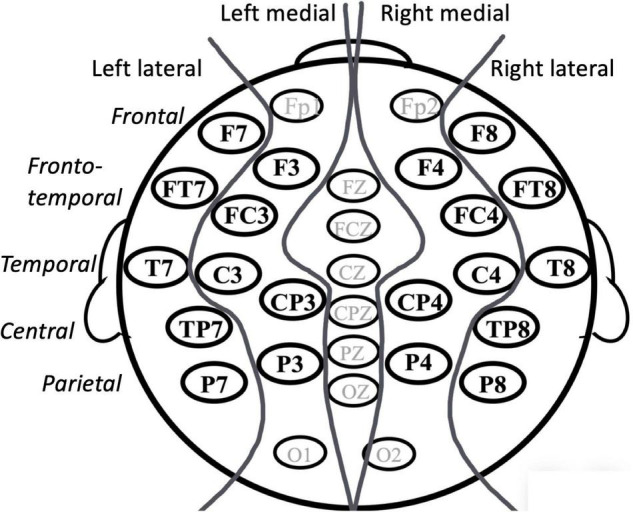
Indicating factors and levels for statistical analyses of ERP effect (cf. Statistical Analyses of Event-Related Potential Effects).

Mean amplitude was measured in time windows chosen in reference to earlier studies and visual inspection of individual waveforms: 300–500, 500–700, and 700–900 ms. The data were subjected to a repeated-measures ANOVA with four within-subject factors, typicality (typical/atypical), hemisphere (right/left), lateral position (lateral/medial), and anterior/posterior position aka ant/post (frontal/frontotemporal/temporal/central/parietal; refer to Figure 3), and one between-subjects factor, language group (Swedish/German/English). Interactions with typicality in the omnibus analyses (*p* < 0.1) were followed up to isolate the location of these interactions in a step-down fashion, correcting for multiple analyses (Bonferroni correction). In addition, as we had *a priori* hypotheses about group differences, the effects in each language group were analyzed separately. The Greenhouse-Geisser correction was applied to all measures with more than two levels. Corrected *p*-values and uncorrected degrees of freedom are reported. All effects with *p*s < 0.1 will be presented. We report partial eta-squared (η*_*p*_*^2^) as a measure of the strength of the reported effects, with η*_*p*_*^2^ of 0.01–0.05 representing a small effect, η*_*p*_*^2^ = 0.06–0.13 a medium effect, and η*_*p*_*^2^ equal to or above 0.14 a large effect.

All trials were included to allow for variability in effects with ratings. To further explore potential relationships between ERP effects and appropriateness ratings and for learners with Swedish proficiency (Swedex), Pearson’s correlation analyses between mean difference amplitude (the effects of typical verb use subtracted from those to atypical verb use) were calculated for each participant, across all sites in each time window. These statistical analyses enable us to better separate and distinguish the effects of formal proficiency in Swedish and specific proficiency of placement verbs from CLI (i.e., group differences).

## Results

### Appropriateness Ratings

**FIGURE 4:**
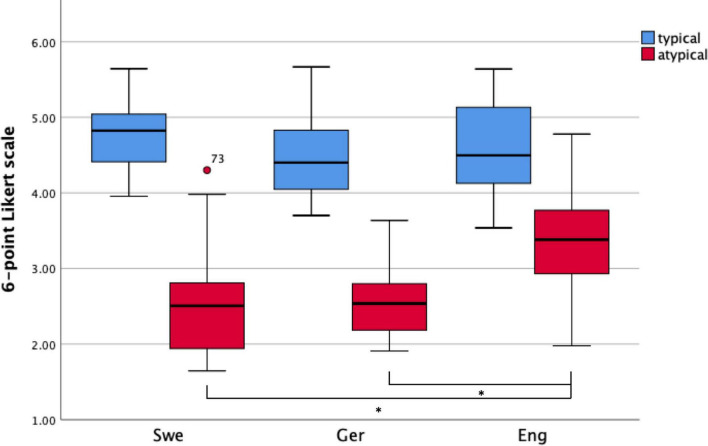
Appropriateness ratings on a 6-point Likert scale (blue, typical verb use; red, atypical verb use) for each language group, i.e., Swedish (Swe), German (Ger), and English (Eng). The solid line within the box represents the average, while the whiskers represent quartiles 1 and 4. The ring above quartile 1 in the Swedish group indicates an outlier (more than two standard deviations higher than the average). These boxplots visualize that the English group does not make as large a difference between typical and atypical verb use as the other two groups. *p < 0.05.

Figure 4 presents the results of appropriateness ratings across typical and atypical verb use and language groups. The analyses showed a main effect of typicality (with a very large effect size), and a significant interaction between typicality and language group (with a large effect size). Planned simple contrasts showed that the group differences were limited to ratings of atypical verb use when comparing Swedish native speakers or German learners’ ratings with those of English learners (Table 3), such that English learners tended to rate atypical verb use as more appropriate than Swedish native speakers and German learners.

**TABLE 3:** Repeated measures ANOVA and simple contrasts (paired *t*-tests) of appropriateness ratings with typicality.

	*df*	*F*	*t*	*p*	η*_*p*_*^2^
*Typicality*	1, 54	410.60		<0.001	0.88
*Typicality × language*	2, 54	12.00		<0.001	0.31
*Atypical*					
Swedish-English	34		–3.44	0.002	
German-English	39		–4.20	<0.001	

*The top of the table displays the results of the ANOVA with F-values and effect size, the bottom displays the significant results of the simple contrasts with t-values. Typical and atypical verb use as rated on a 6-point Likert scale. Planned contrasts comparing each groups’ rating of typical and atypical verb use. Only significant effects displayed.*

### Event-Related Potentials

**FIGURE 5:**
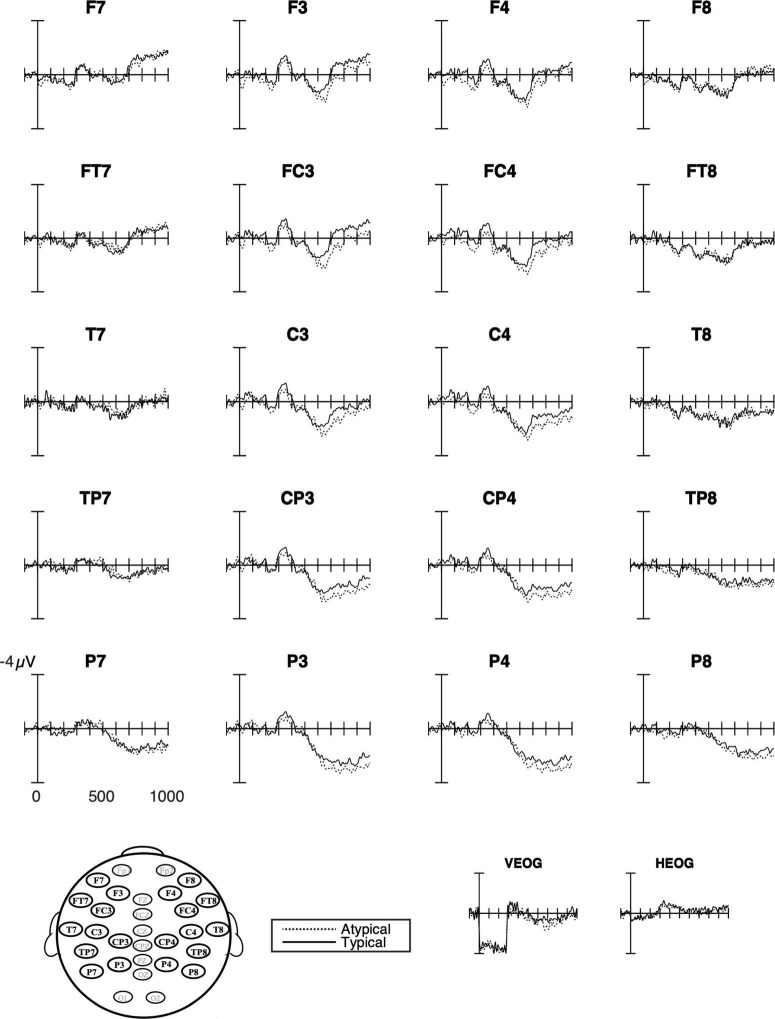
Grand average ERPs for all groups combined at all analyzed sites. The response to the typical verb use (number of trials in the bin, *n* = 1,504) is shown as solid lines and the response to the atypical verb use (*n* = 1,504) as dashed lines. Waveforms suggest left anterior negativity that did not differ from typicality. The P600 that was evident for both verbs posteriorly showed an effect of typicality that was strongest over medial sites. The negative is plotted upward.

While there were no main effects of the language group, the interactions between language group, hemisphere, and typicality approached significance in all time windows (Figure 5 and Table 4, and below).

**TABLE 4:** *F*-values for omnibus and follow-up analyses of ERP effects to Swedish placement verbs.

			300–500 ms		500–700 ms		700–900 ms	
	Variables	*df*	*F*	η*_*p*_*^2^	*F*	η*_*p*_*^2^	*F*	η*_*p*_*^2^
	Typicality × Lateral	1, 54			4.03^†^	0.07	6.98*	0.11
	Typicality × Lateral × Ant/post	4, 216			2.43^†^	0.04	2.37^†^	0.04
	Typicality × Hemisphere × Group	2, 54	2.87^†^	0.10	2.93^†^	0.10	2.73^†^	0.09
Lateral	Typicality × Group	2, 54					2.77^†^	0.09
	Typicality × Hemisphere × Group	2, 54			2.70^†^	0.09	2.54^†^	0.09
Medial	Typicality	1, 54					3.98^†^	0.07
	Typicality × Hemisphere × Group	2, 54			2.73^†^	0.09	2.42^†^	0.08

*Typicality (placement verb, typical/atypical), Hemisphere (left/right), Lateral (lateral/medial), Ant/post (anterior/posterior channels, up to 5 levels), Group (Swedish/German/English). Only licensed follow ups are performed and reported.*

*p < 0.05, ^†^p < 0.10.

Visual inspections of waveforms for typicality combining all three groups suggested a posterior positivity that was strongest over medial sites in the later time windows (Figure 5). The statistical analyses confirmed this P600 effect and showed an interaction with laterality from 500 ms throughout the analyzed epoch (Table 4). This interaction was only marginally significant at 500–700 ms (*p* = 0.05). There was some indication of this medial positivity being stronger over frontal sites. However, the interactions with laterality and anterior-posterior were only marginally significant (500–700 ms, *p* = 0.081; 700–900 ms, *p* = 0.086).

The interactions with hemisphere and language group, which were also marginally significant in all time windows (300–500 ms, *p* = 0.066; 500–700 ms, *p* = 0.062; 700–900 ms *p* = 0.074), suggested different distribution and polarity of the ERP effects to typicality (Figure 5) that will be presented below in analyses for each group. All effect sizes except two were of medium size (Table 4). Follow-up analyses of the interactions with laterality failed to reach significance.

#### Native Swedish Speakers

**FIGURE 6:**
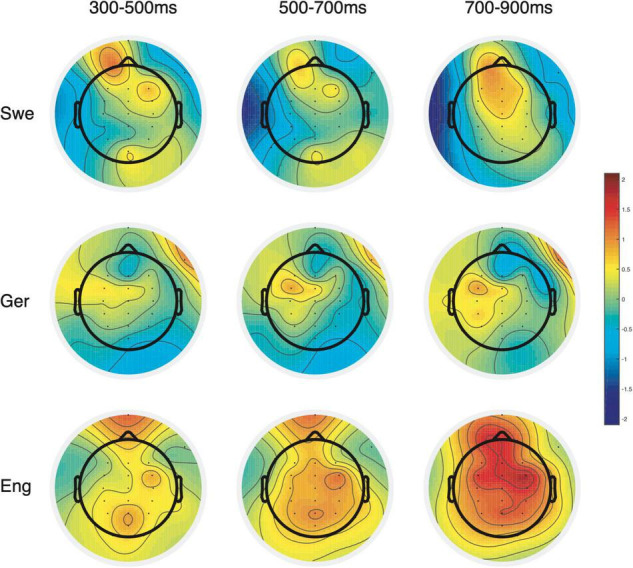
Topographic maps of the ERP effects on the usage of Swedish placement verbs. Columns represent time windows used in statistical analyses of the ERP effects, while rows indicate the effects in Swedish native speakers (Swe), German learners (Ger), and English learners (Eng). These visualizations of the ERP effects show how the effects in German learners are more similar to Swedish native speakers than English learners.

**FIGURE 7:**
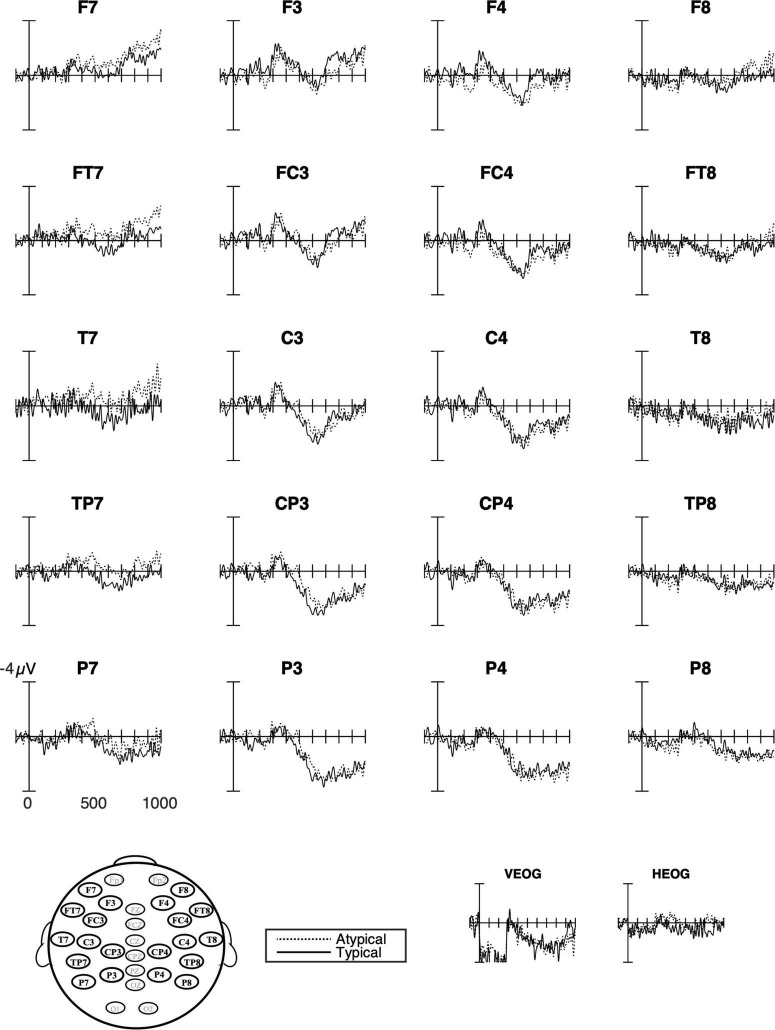
Grand average ERPs for Swedish native speakers. The response to typical verb use (number of trials in the bin, *n* = 429) as solid lines and to atypical verb use (*n* = 428) as dashed lines. The waveforms suggest a left lateral negativity that was strongest for the atypical use of placement verbs followed by a positivity that did not vary with typicality.

Visual inspections of the waveforms suggested lateral negativity that was strongest over the left hemisphere sites and continuing 300–900 ms and extending over the left medial sites for the native speakers of Swedish. However, the statistical analyses captured only lateral negativity at 700–900 ms, which was indicated by significant interactions with typicality and laterality (Figures 6, 7 and Table 5). In the same time window, the marginally significant interaction (*p* = 0.090) with laterality and anterior-posterior suggested that the lateral negativity was stronger over frontal sites. However, there were no significant main effects of typicality over any subset of electrode sites.

**TABLE 5:** *F*-values for follow up analyses of group ERP effects to Swedish placement verbs.

			500–700 ms		700–900 ms	
Group	Variables	*df*	*F*	η*_*p*_*^2^	*F*	η*_*p*_*^2^
Swedish	Typicality × Lateral	1, 15			5.26*	0.26
	Typicality × Lateral × Ant/post	4, 60			2.46^†^	0.14
German	Typicality × Hemisphere	1, 20			3.48^†^	0.15
English	Typicality	1, 19	3.02^†^	0.14	7.52*	0.28
	Typicality × Lateral	1, 19			3.44^†^	0.15

*No significant effects 300–500 ms, thus not included as a column. Typical (placement verb, typical/atypical), Hemisphere (left/right), Lateral (lateral/medial), Ant/post (anterior/posterior channels, up to 5 levels).*

*p < 0.05, ^†^p < 0.10.

#### German Learners of Swedish

**FIGURE 8:**
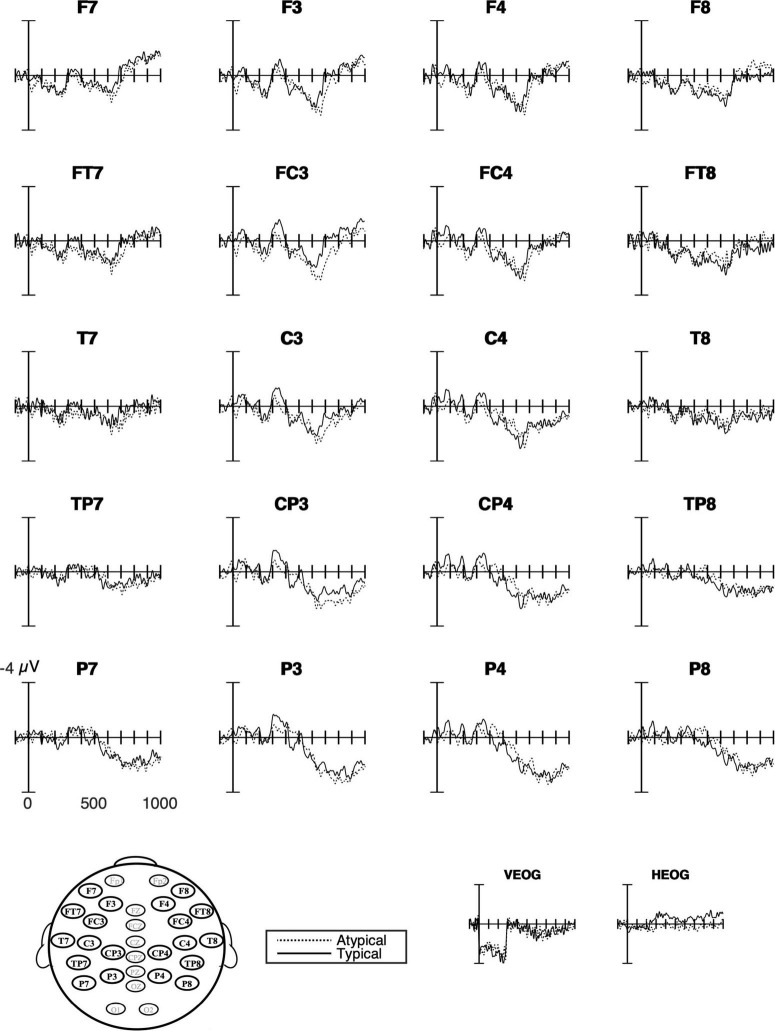
Grand average ERPs for German learners. The response to typical verb use (number of trials in the bin, *n* = 546) as solid lines and to atypical verb use (*n* = 551) as dashed lines. The waveforms suggest a right lateral anterior negativity (F8 and FT8) for the atypical use of placement verbs and over posterior sites and a right negativity (e.g., P4) followed by a positivity strongest over left medial parietal sites.

The ERP waveforms for German learners of Swedish (Figures 6, 8) suggested a late right lateral negativity over anterior sites and a medial positivity (P600) in combination with a right posterior negativity. However, no more than an interaction between typicality and hemisphere approached significance and only at 700–900 ms (*p* = 0.077; Table 5). Follow-up analyses of these interactions failed to reach significance.

#### English Learners of Swedish

**FIGURE 9:**
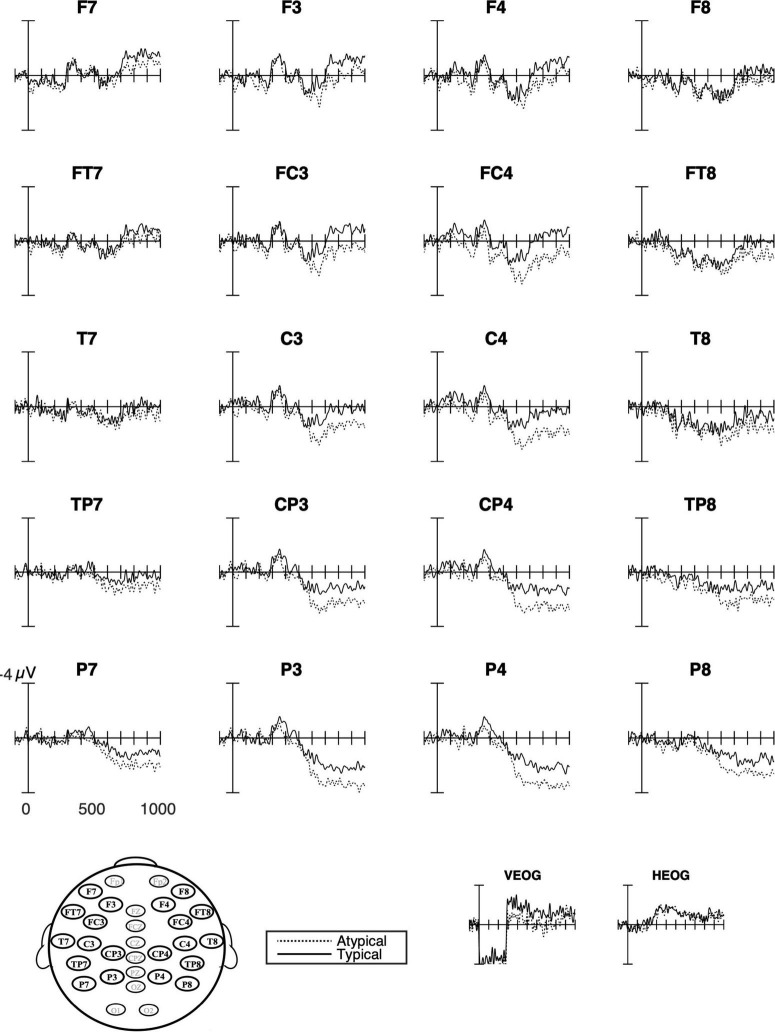
Grand average ERPs for English learners. The response to typical verb use (number of trials in the bin, *n* = 529) as solid lines and to atypical verb use (*n* = 525) as dashed lines. The waveforms suggest a broadly distributed medial positivity for the atypical use of placement verbs.

The waveforms for English learners of Swedish (Figures 6, 9) showed a strong medial positive effect (P600) for typicality. This was confirmed in the statistical analyses where the main effect of typicality approached significance at 500–700 ms (*p* = 0.099) and was significant in the following time window (Table 5). The interaction with laterality that was marginally significant (*p* = 0.079) in the last time window indicated the positivity as being stronger medially in this time window only. Similar to the results from the two other groups, all follow-up analyses of interactions failed to reach significance.

### Relationships Between Event-Related Potential Effects and Appropriateness Ratings

The relationships between ERP effects and appropriateness ratings for typical and atypical verb use are not straightforward. A high rating for typical verb use should be related to a small ERP effect (i.e., no or weak correlation as there would not be an ERP effect), indicating the ease of processing of this verb use, whereas a low rating for typical verb use should therefore be related to a larger ERP effect (i.e., a moderate or strong correlation). Since an ERP effect can be either positive or negative, this complicates the interpretation of the results. Next, we attempt to interpret the results presented in Table 5.

Bivariate correlations (Pearson’s) for native Swedish speakers revealed a medium-strength negative correlation between appropriateness ratings for atypical verb use and the ERP effect at 300–500 ms (Table 6), indicating a stronger positivity with lower ratings. The positive correlation between appropriateness ratings for typical verb use and the ERP effect at 700–900 ms was marginally significant (*p* = 0.073) and indicated a stronger positivity with higher ratings. That is, in both cases, ratings that agreed with the expectation (lower for atypical and higher for typical) were associated with a stronger positive effect in native Swedish speakers. For learners, the correlations with the ERP effects were changing as a function of Swedish proficiency, as measured by the Swedex test (Table 6), such that, with higher general Swedish proficiency, a stronger positive effect was more likely at 500–900 ms.

**TABLE 6:** Correlations amplitude of ERP effects with appropriateness ratings.

		ERP effect across all electrode sites		
		300–500 ms	500–700 ms	700–900 ms
Swedish	Appropriateness rating: Typical			*r* = 0.46^†^
	Appropriateness rating: Atypical	*r* = –0.54*		
Learners	Swedex		*r* = 0.34*	*r* = 0.38*

*Appropriateness rating of Typical and Atypical verb use in the online questionnaire. ERP effect: Difference amplitude (atypical-typical) averaged over all sites in each separate time window.*

*p < 0.05, ^†^p < 0.10.

## Discussion and Conclusion

In this study, we manipulated the typicality of verb use to match pictures depicting placements of objects on a flat surface and recorded appropriateness ratings and ERPs in native Swedish speakers and two learner groups whose L1 placement verb categories either shared semantic characteristics with the target language (German learners) or not (English learners). In all three groups, we found higher ratings for typical verb use (*sätta, “set”* and *ställa “stand”* with symmetrical and asymmetrical objects resting on their functional base, and *lägga* “lay” with objects without a functional base or objects not resting on their functional base) than for atypical verb use. In addition, all three groups showed effects of verb use in their processing as measured with ERPs in the form of interactions with typicality that were either significant or approaching significance. Moreover, as predicted, both ratings and processing in the learner groups showed some evidence of CLI. Specifically, English learners, whose L1 verb semantics do not share semantic characteristics with Swedish, differed both from native Swedish speakers and from German learners, whose L1 verb semantics share some characteristics with Swedish and who differed less from Swedes. Although some of these results only approached significance, all results were in the expected direction. Next, we discuss the findings in more detail, first for appropriateness ratings, followed by the ERP results for native Swedish speakers and then learners, all with a focus on CLI.

Even with the restricted number of items and a small number of participants, all groups showed higher acceptability ratings for typical verb use than for atypical verb use, as expected. That is, the learner groups had clearly learned something about Swedish placement verb semantics. However, there was considerable variation within each group. This was true also for the native Swedish speakers and especially for ratings of atypical verb use, such as e.g., *sätta* with an object without a base. That is, the acceptability of placement verb use is not entirely clear-cut even for native speakers of Swedish for certain placement event scenarios, especially in cases of atypical verb use as indicated by the large variability in the appropriateness ratings (typical: *M* = 4.8, *SD* = 0.5; atypical: *M* = 2.5, *SD* = 0.8). We do not fully understand the source of this variability; it could be related to regional differences in placement verb usage in Sweden, to the potential influence of other languages learned (all Swedes will also know English and often a third language as well), or to the nature of the stimuli or the task. These are all issues that must be further examined.

Behavioral group differences were restricted to atypical verb use, where English learners showed higher acceptability ratings than native Swedish speakers and German learners. More specifically, however, the English learners’ mean ratings of atypical verb use (*M* = 3.4, *SD* = 0.7) were at chance level (3.5). That is, they rated such items as neither acceptable nor unacceptable. Therefore, all learners could identify typical verb use, but atypical verb use was easier to identify if the first language shared semantic characteristics with the target language, with a consideration of the physical properties of the object being placed. Since learners were matched on the age of acquisition, length of exposure, and formal proficiency in Swedish, the differences in learners’ appropriateness ratings of these highly frequent verbs seem only to be explainable by CLI from the L1. However, to ensure that this is the case, we should investigate ratings and processing of L1 categories as well. In addition, it is worth noting that both learner groups must restructure their semantic categories—the English learners must split their *put* category into three, and the German learners must restructure a native category with two verbs into three and base the choice of verb on functional base rather than the object’s extension. The particular challenges in rating atypical events echo previous findings concerning difficulties in rejecting ungrammatical items in judgment tasks (cf. Sorace, 1996). Overall, these findings replicate reported crosslinguistic effects on the production of Swedish placement verbs (Viberg, 1998, 1999) and in placement verb comprehension as shown, for instance, by anticipatory looks to objects following placement verbs in Dutch (van Bergen and Flecken, 2017).

Turning to the ERP results, in native Swedish speakers’ ERP waveforms (Figure 7), there was a significant negative ERP effect for typicality as defined in the study, indicating that the typical verb use was more expected than the atypical verb use. Moreover, the lateral distribution of this negativity could be an indication of an overlap with a medial positivity as suggested by the correlations with different amplitude of the effects and appropriateness ratings. However, this positivity did not differ between typical and atypical verb use, suggesting that native speakers reanalyzed the verbs whether the use was typical or not. It is possible that this positivity to both typical and atypical verb use was elicited by the task since each placement event was followed by a request for a forced-choice button press indicating acceptability or not. However, it could also be related to individual variability in verb use and to our predefined coding of what is typical/atypical verb use based on ratings from a previous study (Andersson and Gullberg, 2016). That is, we know that the use of *sätta/ställa* in particular shows some individual variation, which could result in a mismatch between individual preferences and expectations, even if both verbs were considered to be typical in our analyses. Any such mismatch would result in increased variability in the ERP effects for typical verb use.

As for L2 learners, despite the mixed results from the native Swedish speakers, we can still assess whether learners whose L1s share or do not share verb semantics with the target language process placement events differently as a function of that distinction. As expected, there were more similarities in the ERP waveforms between German learners and Swedish native speakers than between English learners and Swedish speakers. Just as for Swedes in the current study, the German waveforms and topographic maps (Figures 6, 8) suggested a right posterior negativity and a left medial positivity. However, importantly, the statistical analyses only approached significance, even if they showed a large effect size. In contrast, and as expected, English learners differed from both native Swedish speakers and German learners. More specifically, English learners showed a strong positive effect of typicality (i.e., stronger positivity for atypical than typical verb use) that extended over all sites but was strongest over medial sites. There were no indications of any negativities. It is possible that the average ERP effects indicate, on an item level, that German learners process L2 Swedish in some cases as native Swedish speakers do and in other cases as English learners, and thus the effects are attenuated in the average waveforms. Due to unforeseen obstacles to participant recruitment, the group sizes do not make item-based analyses viable. However, in contrast and importantly, our analyses suggest that the English learners’ process verb use differently from both of the other groups. The findings from this exploratory study are in line with results from a study of Swedish word order processing with the same language groups (German and English learners; Andersson et al., 2019). Although the effects were of different distribution and polarity, they showed the same pattern as here, namely that native Swedish speakers and English learners differed the most, while German learners were somewhere in between and more similar to Swedish speakers.

Contrary to our hypotheses, the effect of typicality set in earlier in English learners than in German learners and Swedish native speakers. The later effects in the Swedish and German groups could be due to an overlap between an early negativity and an early positivity, which cancel each other out in the group analysis, but again, with the limited number of participants, this could not be clarified in our analyses. The stronger positivity for atypical verb use in English learners in combination with the higher appropriateness ratings possibly reflects uncertainty in the ratings for atypical verb use in this group (Dröge et al., 2016). Thus, these learners are more certain about typical than atypical verb use, suggesting that they have acquired the verb semantics at least in part. In addition, although we replicated findings from previous studies of semantic processing, in that the correlational analyses indicated a stronger effect with higher general proficiency in the second language (e.g., FitzPatrick and Indefrey, 2010; Midgley et al., 2010), this effect was positivity in response to the verb rather than the N400 typically elicited in semantic processing studies. The presence of positivity rather than the N400 suggests that the processing of placement verbs is more complex than the semantic violations typically used in studies of semantic processing in L1 and L2.

In conclusion, this study has two specific outcomes. First, even when fine-grained verb semantics differ between the L1 and the L2, learners can develop sensitivities to such distinctions and make conscious judgments about them offline (cf. Tokowicz and MacWhinney, 2005), especially in cases where scenarios represent typical verb use in the target language. Second, the semantic similarity between the L1 and the L2 affects offline but to some extent also online performance. Learners with similar semantic distinctions in their L1 (German learners) both judge and seem to process L2 verbs more similarly to native speakers (of Swedish) than learners whose L1 lack similar semantic distinctions (English learners). Importantly, in comparison to previous studies of semantic processing, the results from this study suggest that the processing of placement verb semantics can be qualitatively different depending on language background and that crosslinguistic differences in fine-grained verb semantics affect the L2 both offline and online.

## Data Availability Statement

The datasets presented in this study can be found in online repositories. The names of the repository/repositories and accession number(s) can be found below: The Open Science Framework https://osf.io/3h86e/.

## Ethics Statement

The studies involving human participants were reviewed and approved by the Regional Ethics Board in Linköping 2017/607-31. The patients/participants provided their written informed consent to participate in this study.

## Author Contributions

AA developed the stimuli, the ERP paradigm, and analyzed all data. Both authors contributed to the conception and experimental design of the study and contributed to the interpretation of the results, the writing of the manuscript, and approved the final version of the manuscript for submission.

## Conflict of Interest

The authors declare that the research was conducted in the absence of any commercial or financial relationships that could be construed as a potential conflict of interest.

## Publisher’s Note

All claims expressed in this article are solely those of the authors and do not necessarily represent those of their affiliated organizations, or those of the publisher, the editors and the reviewers. Any product that may be evaluated in this article, or claim that may be made by its manufacturer, is not guaranteed or endorsed by the publisher.

## Footnotes

1 The field of language assessment and scholarship on proficiency tests is vast with annual conferences, a dedicated journal, *Language Testing*, and an international organization, the International Language Testing Association (ILTA). The work in the field indicates that standardized tests are often at best proxies for holistic language skills. However, such tests do allow for some comparisons to be made across groups, which is why they are included.
