# Supplementary material for: First Language Matters: Event-Related Potentials Show Crosslinguistic Influence on the Processing of Placement Verb Semantics
Source: Front Psychol. 2022 Jul 7;13:815801. doi: 10.3389/fpsyg.2022.815801 (PMC9301051; doi:10.3389/fpsyg.2022.815801)
Supplement: Supplementary file 1 [file Data_Sheet_1.docx]

Appendix A

Each of the following type of labelled objects were photographed and presented as in Figure 1 and manipulated as according to Table 2. Note that there were several objects of one type, for example balls and glasses that differed in one of the variables (symmetry, base, and/or animate for fillers). For access to all images please refer to <https://osf.io/3h86e/>

Symmetric without base

Lime

Melon

Potato

Persimmon

Cake

Paper ball

Ball of yarn

Orange

Grapefruit

Golf ball

Mango

Nut

Passionfruit

Swede

Ball (three different types)

Chewing gum ball

Asymmetric without base

Egg

Avocado

Ball (American football)

Yarn

Shark egg

Kiwano

Kiwi

Toy

Carrot

Mussel

Pear

Easter egg

Ring

Sweet potato

Asparagus

Spiral

Sponge

Zuchini

Symmetric with base

Dice

Cube

Loaf of bread

Wooden block

Jar

Roll of string

Lid

Pot

Bun

Duplo piece

Candy

Asymmetric with base

Pineapple

Card box

Avocado

Deodorant

Bottle

Glass

Roll of paper towels

Camera

Tin can

Flowerpot

Box

Lipstick

Candle

Nail polish

Grater

Brush for shaving

Bowl

Toilet paper roll

Symmetric inanimate

Car

Sieve

Jug

Tower

Car

Flowerpot with legs

Bucket

Wine glass

Asymmetric inanimate

Bench

Boat

Car

Table

Toaster

Bus

Locomotive

Airplane

Rocket

Train

Tractor

Symmetric animate

Old woman

Minion

Chicken

Hedgehog

Rabbit

Toy

Asymmetric animate

Caterpillar

Duck

Barbie

Doll

Tomas the tank engine torch

Pig

Old man

Horse

Cow

Teddy bear

Pony

Fox

Odd positions

Banana

Car

Brie cheese

CD

Chocolate

Pot

Cucumber

Hole punch

Cake

Button

Laptop

Tea candle

Key

Plate
